# The APOE locus is linked to decline in general cognitive function: 20-years follow-up in the Doetinchem Cohort Study

**DOI:** 10.1038/s41398-022-02258-5

**Published:** 2022-11-29

**Authors:** M. Liset Rietman, N. Charlotte Onland-Moret, Astrid C. J. Nooyens, Dorina Ibi, Ko Willems van Dijk, Leonard Daniël Samson, Jeroen L. A. Pennings, Maarten Schipper, Albert Wong, Annemieke M. W. Spijkerman, Martijn E. T. Dollé, W. M. Monique Verschuren

**Affiliations:** 1 National Institute for Public Health and the Environment, Bilthoven, The Netherlands; 2 Julius Center for Health Sciences and Primary Care, University Medical Center Utrecht, Utrecht University, Utrecht, The Netherlands; 3 Department of Human Genetics, Leiden University Medical Center, Leiden, The Netherlands; 4 Department of Internal Medicine, Division of Endocrinology, Leiden University Medical Center, Leiden, The Netherlands

**Keywords:** Predictive markers, Genetics

## Abstract

Cognitive decline is part of the normal aging process. However, some people experience a more rapid decline than others due to environmental and genetic factors. Numerous single nucleotide polymorphisms (SNPs) have been linked to cognitive function, but only a few to cognitive decline. To understand whether cognitive function and cognitive decline are driven by the same mechanisms, we investigated whether 433 SNPs previously linked to cognitive function and 2 SNPs previously linked to cognitive decline are associated with both general cognitive functioning at baseline and general cognitive decline up to 20-years follow-up in the Doetinchem Cohort Study (DCS). The DCS is a longitudinal population-based study that enrolled men and women aged 20–59 years between 1987–1991, with follow-up examinations every 5 years. We used data of rounds 2–6 (1993–2017, *n* = 2559). General cognitive function was assessed using four cognition tests measuring memory, speed, fluency and flexibility. With these test scores, standardized residuals (adjusted for sex, age and examination round) were calculated for each cognition test at each round and subsequently combined into one general cognitive function measure using principal component analyses. None of the 435 previously identified variants were associated with baseline general cognitive function in the DCS. But rs429358-C, a coding apolipoprotein E (APOE) SNP and one of the variants previously associated with cognitive decline, was associated with general cognitive decline in our study as well (*p*-value = 1 × 10^−5^, Beta = −0.013). These findings suggest that decline of general cognitive function is influenced by other mechanisms than those that are involved in the regulation of general cognitive function.

## Introduction

For healthy aging it is essential to maintain optimal cognitive function throughout the course of life. Preserving good cognitive function is important to remain self-reliant and to prevent or postpone cognitive impairment and dementia [1]. Decline in cognitive function is part of the normal aging process [2], but there is large inter-individual heterogeneity in the rate of decline. Moreover, accelerated cognitive decline is a predictor of dementia and mortality [3–5]. Multiple risk factors that negatively affect cognitive function and cognitive decline are known, such as lifestyle factors (physical inactivity, smoking, unhealthy diet), metabolic factors (hypertension, obesity, diabetes mellitus), and a lower educational level [6–9]. In addition to these (partly) modifiable factors, genetic factors play an important role. For example, it has been shown that apolipoprotein E (APOE) ε4 carriers have accelerated cognitive decline, while APOE ε2 carriers have decelerated cognitive decline compared to ε3 carriers in middle aged and older adults [10, 11]. In addition, it has been shown that already in childhood APOE ε4 affects cognitive performance [12].

Studying the role of genetic factors, through for example genome-wide association studies (GWASs), could disclose underlying biological mechanisms affecting cognitive health. In addition, it may be of even greater value to identify single nucleotide polymorphisms (SNPs) associated with a decline in cognitive function, rather than with cognitive functioning at a single point in time. This may reveal specific mechanisms behind cognitive decline, preceding cognitive impairment and dementia. In addition, genetic markers can also help identify people who are at risk of (accelerated) cognitive decline and possibly postpone or reduce cognitive decline, for example by increasing the cognitive reserves [13].

Numerous SNPs have been linked to cognitive function. In a recent GWAS by Davies and Lam et al., including over 300,000 participants, 434 independent SNPs (i.e. SNPs with a *p*-value of ≤5 × 10^−8^ and *r*^2^ < 0.6) in 148 genomic loci were associated with general cognitive function cross-sectionally [14]. Only a few SNPs have been linked to cognitive decline in GWASs, possibly due to the limited number of cohort studies in which cognitive functioning is repeatedly measured. These studies showed that APOE is associated with cognitive decline in people with different genomic backgrounds [15–17]. Surprisingly, the APOE locus was not amongst the associated loci in the GWAS by Davies and Lam et al. on cross-sectional cognitive function [13]. This raises the question whether different genetically determined pathways influence the level of cognitive function and the rate of cognitive decline. Therefore, we investigated whether the recently identified independent SNPs by Davies and Lam et al. [14] along with two APOE SNPs, were associated with general cognitive functioning at baseline and general cognitive decline in the Doetinchem Cohort Study (DCS) over an extended period of time (up to 20-years follow-up) and with up to five repeated cognition measurements in older adults (*n* = 2559).

## Materials and methods

### Cohort

The DCS is a longitudinal population-based cohort study including 7769 men and women aged 20–59 years living in Doetinchem between in 1987–1991 (round 1). Adults who participated in the first round were invited for follow-up examinations in 1993–1997 (round 2, *n* = 6117, mean age: 46 years), 1998–2002 (round 3, *n* = 4918, mean age: 51 years), 2003–2007 (round 4, *n* = 4520, mean age: 56 years), 2008–2012 (round 5, *n* = 4018, mean age: 60 years), and 2013–2017 (round 6, *n* = 3438, mean age: 64 years). Response rates were 75% or higher in all rounds. The design of this study has previously been described in more detail [18, 19]. All participants gave written informed consent in each round. The study was approved by the Medical Ethics Committee of the Netherlands Organization of Applied Scientific Research and the Medical Ethics Committee of the University of Utrecht according to the guidelines of the Helsinki Declaration.

#### Measurements

Weight (kg), height (cm), waist circumference (cm), and systolic and diastolic blood pressure (mmHg) were measured according to standard protocols [19]. BMI was calculated as weight divided by height squared (kg/m^2^). Obesity was defined as having a BMI ≥ 30 kg/m^2^. Standardized questionnaires were used to obtain data on education level (low, intermediate, high), smoking status (never smoker (including former smokers), current smoker), alcohol consumption (never, stopped consuming, <1 glass/week, 1 or more glasses/week), physical activity (categorized using the Cambridge Physical Activity Index; inactive, moderately inactive, moderately active, active) [20], and self-reported health (poor, fair, good, very good, excellent). Education level was measured as the highest level reached during follow-up and categorized into low (intermediate secondary education or less), intermediate (intermediate vocational and higher secondary education) and high (higher vocational education or university). Participants who were physically inactive or moderately inactive were defined as being physically inactive.

Cognitive function was assessed in rounds 2–6 using a neuropsychological test battery among participants aged 45 years and older by trained personnel following a standardized protocol. General cognitive functioning was measured using four tests assessing four domains: memory function, information processing speed, verbal fluency and cognitive flexibility. These four tests were the 15 Words Verbal Learning Test (VLT) (number of correct words on the delayed recall) [21], the Letter Digit Substitution Test (total of correct answers) [22], the Word Fluency Test (number of correct animals) [23], and the Stroop Color–Word Test (card III, i.e. total time needed for the interference test) [24]. The cognitive tests have previously been described in more detail [25].

**Fig. 1:**
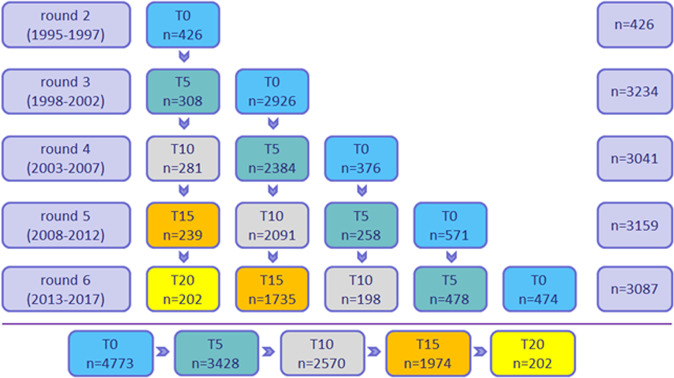
Overview of cognition measurements (T0-T20) through rounds 2–6 in the Doetinchem Cohort Study. This figure shows the number of participants with one or more cognition measurements (from T0 up to T20 (time in years)) through rounds 2–6 of the Doetinchem Cohort Study (DCS) during the 20-year follow-up. *Note:* This figure includes all participants with cognition measurements in the DCS. In the present study, part of these participants were excluded (see Supplementary Fig. 1). T0 = blue, T5 = green, T10 = gray, T15 = orange, T20 = yellow.

The STROOP test was log-transformed. The other tests had a normal distribution. The first cognition measurement of a study participant was considered the baseline measurement, i.e. timepoint zero (T0). Since cognition measurements started when participants had reached the age of 45 and was introduced half-way in round 2 of the DCS, T0 was not confined to a particular round. Most participants had their T0 measurement in round 3, but there were also participants who had their T0 measurement in round 2, 4, 5 or 6 (Fig. 1). Timepoints range from T0-T20 with 5-year intervals.

### Genotyping, quality control and imputation

Genomic DNA was isolated from venous blood samples of 5088 individuals at the Dutch National Institute for Public Health and the Environment, and genotyped in the HUman GEnomics Facility (HUGE-F) Rotterdam using the Illumina Infinium Global Screening Array-24 Kit (GSA) (Illumina Inc., San Diego, California, United States of America) [26]. The R package GenABEL 1.8-0 [27], was used to perform the quality control for both participants and genetic variants.

Participants were excluded if (Supplementary Fig. 1): (1) there was a sex mismatch (*n* = 45), (2) samples were duplicates (*n* = 18) or monozygotic twins (*n* = 1) (one individual per pair), (3) heterozygosity rate was high (false discovery rate (FDR) < 1%) (*n* = 37), (4) the sample call rate was <95% (*n* = 20), and (5) participants were widely diverged (i.e. being genetically distant based on visual inspection of a genomic principal component (gPC) plot) regarding their genetic background based on the first two gPCs that were constructed using a kinship matrix, in two steps (*n* = 114). First, the more distant participants compared to the group as a whole were excluded. Next, as a single iterative step new gPCs were generated in the remaining sample population and additional participants were removed.

Genetic variants were excluded when: (1) minor allele frequencies (MAF) were <1/(2*5088) (*n* = 5088, this is the population before quality control), i.e. the chance of finding the allele once in the study population, thus representing monomorphic variants (*n* = 109129), (2) genotype call rates were <95% (*n* = 8005), (3) variants were not in Hardy-Weinberg equilibrium (FDR < 0.2) (*n* = 0), and (4) X-linked markers were likely to be autosomal (*n* = 421). Subsequently, the HRC-1000G-check-bim.pl script from Rayner [http://www.well.ox.ac.uk/~wrayner/toolsHRC-1000G-check-bim.plscript] was used for quality control and to convert the Plink genotype data [28] to separate VCF files per chromosome. This pre-imputation step of quality control filtered additional SNPs based on genotype call rate <98% (*n* = 15013) and Hardy-Weinberg *p* < 10^−6^ (*n* = 0). Finally, genotypes were imputed to the Haplotype Reference Consortium (HRC) panel (version r1.1 2016) [29] with the Michigan Imputation Server [30] using NCBI Genome Reference Consortium Human Build 37. Pre-phasing was performed on the imputation server with Eagle v2.3 [29] and imputation with Minimac3 [31]. After quality control and imputation of the GSA-data, a total of 4853 participants were left for further analyses (Supplementary Fig. 1).

### General cognitive function at baseline and during follow-up

For 4110 participants both genotype and cognition data were available (Supplementary Fig. 1). Before constructing the general cognitive function measure, participants without measurements on all four cognition tests were made missing for that particular round. Participants were excluded when: the previous step resulted in missing values for the cognition tests at T0 (*n* = 45), had cognition measurements at only one time point (*n* = 768), or had a history of stroke (diagnosed or self-reported (*n* = 213)) at any measurement.

Using the four tests, we constructed a measure of general cognitive function as described by Davies and Lam et al. [14] and Trampush et al. [32]. In brief, sex, age and examination round-adjusted standardized residuals were calculated for each cognition test at each round. Next, these four adjusted test scores were combined into one general cognitive function measure using a principal component analysis. In the Supplementary Material, we describe each of these steps in more detail. After all the steps had been taken, also shown in Supplementary Fig. 1, 2559 participants were left to study the associations between SNPs and general cognitive function and decline.

### SNP selection

Davies and Lam et al., identified 11.600 SNPs that were statistically significantly (*p*-value of ≤5 × 10^−8^) associated with general cognitive function cross-sectionally. Of these SNPs, they identified 434 ‘independent’ SNPs (at an *r*^2^ cut-off <0.6 [14]). They used NCBI build 37 as reference, which is the same build we used to impute our data. Since in previous GWASs it was shown that APOE gene variants are associated with age related cognitive decline [15, 16], this locus was also part of our interest. Hence, we added rs429358 (chromosome (chr):base pair (bp) 19:45411941) and rs7412 (chr:bp 19:45412079) to our SNP-dataset. Thus, we selected the 434 ‘independent’ SNPs and the two aforementioned exonic APOE SNPs for our study resulting in a total of 436 SNPs. Genetic variants with an imputation quality (*R*^2^) below 0.4 or a minor allele frequency (MAF) below 0.01 were not considered for analysis in the present study. Since one of the 434 ‘independent’ SNPs had a MAF < 0.01 (rs541507329, chr:bp 1:22428398), the final SNP selection consisted of 435 SNPs based on 433 ‘independent’ SNPs and 2 APOE SNPs.

### Statistical analyses

#### Population characteristics

Descriptive analyses were carried out in RStudio version 1.1.456 [33]. Trajectories of general cognitive function up to 20-years follow-up were visualized using ggplot2 version 3.0.0 [34].

#### Cross-sectional association between SNPs and general cognitive function at baseline

We studied the cross-sectional association between the 435 SNPs (independent variables) and general cognitive function at baseline (T0) (dependent variable). A linear regression model was fitted per SNP and in each model we adjusted for sex, age, and population stratification using the first two gPCs using RVTESTS version 20190205 [35]. We corrected for multiple testing based on the Bonferroni adjustment (i.e. *p*-value is 0.05/435 = 1 × 10^−4^). Hence, a *p*-value < 1 × 10^−4^ was considered statistically significant.

Since it is known that education level strongly influences the level of general cognitive function, but most likely not that of cognitive decline [36, 37] (see also Supplementary Fig. 2), we performed a sensitivity analysis in which we studied the effect of education level in the cross-sectional association between SNPs and general cognitive function, with the linear regression model (for baseline cognitive function) as described above to which we added education level as a covariate.

#### Longitudinal association between SNPs and general cognitive decline

To study the longitudinal association between the 435 SNPs (independent variables) and decline in general cognitive function (T0-T20) (dependent variable) we used LME4 version 1.1–17 [38]. This package can handle missing values, as long as each participant has at least two observations. A linear mixed model was fitted for each SNP and in each model we adjusted for sex, age at baseline, and population stratification using the first two gPCs. In addition, we included time (0–20 years with 5-year intervals, i.e. five time points) into the model, and the interaction terms SNP*time and age at baseline*time. For this model we used a correlated random intercept and slope, since participants with higher cognitive function at baseline (intercept) may have a steeper decline (slope), and vice versa. A *p*-value of 0.1, instead of 0.05, was considered statistically significant since we are now interested in an interaction term instead of a main effect. We corrected for multiple testing based on the Bonferroni adjustment (i.e. 0.1/435 = 2 × 10^−4^) for the interaction term SNP*time. Hence, a *p*-value < 2 × 10^−4^ was considered statistically significant.

#### Longitudinal association between the APOE haplotype group and general cognitive decline

The two APOE SNPs were also assessed as a haplotype, that is, rs429358 and rs7412 were combined to obtain the APOE genotypes (ε2ε2, ε2ε3, ε3ε3, ε2ε4, ε3ε4, and ε4ε4). There were no ε1 carriers present in this study population. Next, the participants were grouped in ε2 carriers (ε2ε2 and ε2ε3), ε3 homozygotes (ε3ε3), and ε4 carriers (ε2ε4, ε3ε4, and ε4ε4). Since the ε4 allele is dominant over the ε2 allele [39], ε2ε4 genotypes were included in the ε4 carriers-group. To study the longitudinal association between the three APOE groups (independent variable with ε3 homozygotes as reference group) and decline in general cognitive function (T0-T20) (dependent variable) we used the same model as used to study the longitudinal association between the 435 SNPs and general cognitive decline (see section above). A *p*-value of 0.1 was considered statistically significant since we were interested in the interaction term APOE haplotype group*time.

#### Polygenic profile score analyses

A polygenic profile score was calculated based on 399 out of the 435 independent SNPs. The palindromic SNPs (*n* = 36) were excluded from these analyses. The polygenic profile score was calculated using PLINK (version 1.90) based on the summary statistics of the 399 independent variants of Davies and Lam et al. [14]. The proportion of explained variance (R²) was calculated using a linear regression model per time point (T0-T20). A separate linear regression model was used to examine the cross-sectional association between the polygenic profile score and general cognitive function at baseline, adjusting for age at baseline, sex, and population stratification using the first two gPCs. To study the longitudinal association between the polygenic profile score and general cognitive decline we used a linear mixed model adjusting for sex, age at baseline, and population stratification using the first two gPCs. In addition, we included time (0–20 years with 5-year intervals, i.e. five time points) into the model, and the interaction terms polygenic profile score*time and age at baseline*time. A *p*-value of 0.1 was considered statistically significant since we were interested in the interaction term polygenic profile score*time.

## Results

### Population characteristics

The study sample consisted of 2559 participants at T0, 2434 at T5, 1832 at T10, 1423 at T15, and 130 at T20. There were 707 participants with two cognition measurements, 556 with three, 1184 with four, and 112 with five cognition measurements, resulting in 8378 observations. Participants did not always have consecutive measurements, meaning that participants could for example have cognition measurements at T0, T10, and T20. The population characteristics at baseline (T0) are summarized in Table 1.

**Table 1:** Population characteristics at baseline (T_0_).

	Characteristics at T_0_
	*N* = 2559
**Socio-demographic factors**	
Sex (men)	47%
Age (years), median (IQR)	53.0 (8.7)
Low education level	41%
**Life-style factors**	
Physically inactive	23%
Current smoker	22%
Alcohol consumption (1 or more glasses/week)	73%
**Anthropometric data**	
BMI (kg/m^2^), mean (SD)	26.3 (3.9)
Obesity (≥30 kg/m^2^)	14%
Waist circumference (cm), mean (SD)	93.9 (11.3)
**Blood pressure**	
Systolic blood pressure (mmHg), mean (SD)	129.2 (17.4)
Diastolic blood pressure (mmHg), mean (SD)	81.6 (10.5)
**Health**	
Poor or fair self-reported health	12%

*BMI* body mass index, *SD* standard deviation, *IQR* interquartile range.

**Fig. 2:**
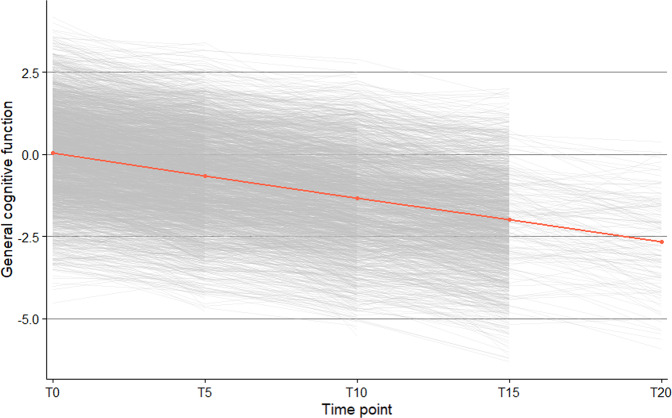
Trajectories of general cognitive function. This figure shows the trajectories (with 95% confidence intervals) of general cognitive function up to 20-years follow-up (*n* = 2559). The general cognitive function measure is based on sex, age and examination round-adjusted standardized residuals. T0-T20 represents time in years. The red dots represent mean general cognitive function at each time point.

Figure 2 shows the trajectories of all 2559 participants for general cognitive function. We also visualized the trajectories of the sex, age and examination round-adjusted standardized residuals of the four individual cognition tests (Supplementary Figs. 4–7).

### Cross-sectional association between SNPs and general cognitive function at baseline

After adjustment for multiple testing, none of the 435 SNPs were significantly associated with general cognitive function at baseline (Supplementary Table 1). The SNP with the lowest *p*-value (*p*-value = 2 × 10^−4^) was rs2782653 located at chr:bp 1:43950265. The alternative allele G was inversely associated with general cognitive function at baseline (Beta = −0.16) compared to the reference allele C.

Additional adjustment for level of education did not change the results (Supplementary Table 1). Supplementary Fig. 2 shows trajectories of general cognitive function stratified by education level up to 20-years follow-up.

### Longitudinal association between SNPs and general cognitive decline

After adjusting for multiple testing, rs429358, one of the two APOE SNPs, was statistically significantly associated with decline in general cognitive function (*p*-value = 1 × 10^−5^, Beta = −0.013) with T as reference allele and C as alternative allele (Supplementary Table 2). Supplementary Fig. 3 shows the trajectories stratified by rs429358 genotype up to 20-years follow-up. None of the other SNPs were significantly associated with a decline in general cognitive function.

### Longitudinal association between APOE haplotype group and general cognitive decline

**Fig. 3:**
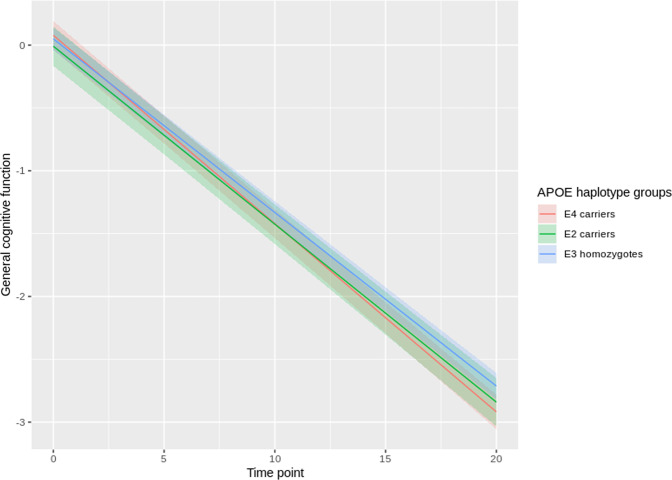
Adjusted trajectories of general cognitive function for the APOE haplotype groups. This figure shows the adjusted trajectories of general cognitive function for three APOE groups (i.e. ɛ2 carriers, ɛ3 homozygotes and ɛ4 carriers) up to 20-years follow-up (*n* = 2559). T0-T20 represents time in years. The trajectories were adjusted for sex, age at baseline, and population stratification using the first two gPCs. In addition, we included time (0–20 years with 5-year intervals, i.e. five time points) into the model, and the interaction terms SNP*time and age at baseline*time.

Table 2 gives an overview of the APOE haplotypes for the total population and for men and women based on rs429358 and rs7412. Supplementary Table 3 shows the APOE haplotypes stratified by education level. There was no significant longitudinal association between the ε2 carriers and ε3 homozygotes (reference category) for general cognitive decline. However, there was a statistically significant longitudinal association between the ε4 carriers and ε3 homozygotes (reference category) for general cognitive decline (*p*-value = 5 × 10^−4^, Beta = −0.012). Figure 3 shows the adjusted trajectories of general cognitive function for the APOE haplotype groups based on this longitudinal association.

**Table 2:** APOE haplotypes for the total population and for men and women separately.

Haplotypes	Total	Men	Women	APOE groups
	*N* = 2559	*N* = 1195	*N* = 1364	
ɛ2/ɛ2	1%	1%	1%	ɛ2 carriers
ɛ2/ɛ3	6%	7%	6%	ɛ2 carriers
ɛ3/ɛ2	6%	6%	6%	ɛ2 carriers
ɛ3/ɛ3	58%	58%	57%	ɛ3 homozygotes
ɛ3/ɛ4	11%	11%	11%	ɛ4 carriers
ɛ2/ɛ4	2%	2%	2%	ɛ4 carriers
ɛ4/ɛ2	1%	2%	1%	ɛ4 carriers
ɛ4/ɛ3	12%	12%	13%	ɛ4 carriers
ɛ4/ɛ4	3%	3%	3%	ɛ4 carriers

### Polygenic profile score analyses

At T0, 0.9% of the variance of general cognitive function is explained by the polygenic profile score (Table 3). At time points T5, T10 and T15, compared to T0, less variance of general cognitive function is explained by the polygenic profile score. At T20, a higher percentage (2.9%) of the variance of general cognitive function is explained by the polygenic profile score compared to the other time points. Further, we found a statistically significant cross-sectional association between the polygenic profile score and general cognitive function (Beta = 0.72, standard error = 0.15, *p*-value = 9.3 × 10^−7^). The longitudinal association between the polygenic profile score and general cognitive decline was not statistically significant (Beta = 0.001, standard error = 0.007, *p*-value = 0.86).

**Table 3:** R² between the polygenic profile score and general cognitive function per time point.

Time point	N	Estimate	*R*^2^	*P*-value
0	2559	0.611	0.0087	2.1 × 10^−6^
5	2434	0.497	0.0058	1.6 × 10^−4^
10	1832	0.445	0.0043	4.9 × 10^−3^
15	1423	0.471	0.0050	7.7 × 10^−3^
20*	130	1.081	0.0293	5.1 × 10^−2^

*The participant rate at T20 is low because the data collection is still in progress.

## Discussion

To understand whether cognitive function and cognitive decline are driven by the same mechanisms, we investigated whether 433 SNPs previously linked to cognitive function and 2 SNPs previously linked to cognitive decline were associated with both general cognitive functioning at baseline and general cognitive decline in the DCS. We found that rs429358-C, one of the APOE SNPs, was associated with long-term general cognitive decline, but not with general cognitive function at baseline. None of the other previously identified SNPs for cognitive function or decline were significantly associated with general cognitive function at baseline, nor with cognitive decline.

One of the strengths of the DCS is that cognitive functioning was repeatedly (up to five measurements) and objectively measured with a standardized, comprehensive and validated neuropsychological test battery in adults over an extended period of time (up to 20-years follow-up), making this a unique cohort to study cognitive aging. In addition, for this study we used data from a single cohort, i.e. the DCS, and used an identical neuropsychological test battery at all examinations resulting in a more homogenous outcome than in a meta-analysis where the included cohorts often use different neuropsychological test batteries. A limitation to this study was the number of included participants. After applying all exclusion criteria there were 2559 participants left for the association analyses. The number of participants may have limited our power since general cognitive function is a complex trait for which a higher number of participants is likely to be needed to gain sufficient power [40].

We were unable to replicate the findings of Davies and Lam et al. [14], i.e. none of their 433 ‘independent’ SNPs were associated with general cognitive function at baseline in our study. However, we did find an association with the polygenic profile score and baseline general cognitive function. There are some explanations for these different results that need to be discussed. First, the age range in the DCS was 45–74 years at baseline, while the age range in the study of Davies and Lam et al. was 16–102 years. Cognitive function changes over the course of life and has an inverted U-shape in which the brain and cognitive functions of adolescents still develop [41], while older adults may experience cognitive decline due to ageing of the brain [42]. In both the DCS study as well as in the study of Davies and Lam et al., adjustment for age was performed. However, since age has such a strong impact on the level of cognitive function, it could still have affected the obtained results differentially. Second, the heterogeneous phenotype could also have influenced our results regarding baseline cognitive function. General cognitive function is a heterogenous outcome in two respects. (1) participants can score differently on the individual cognitive functioning tests while they can have the same score on the overall measure. For example, one participant can have a low memory test score, while another participant can have a low executive functioning test score. This can lead to the same overall score, i.e. general cognitive function, while they score differently on the underlying tests. Between studies, therefore, the average total score may not reflect similar underlying functioning of the participants. (2) frequently, different tests are used to measure cognitive function in different cohort studies. In the DCS, the same neuropsychological test battery was used through all rounds for all participants. In the meta-analyses of Davies and Lam et al., data of multiple cohorts were used in which cognitive function was tested using different test batteries. As a result, phenotypic heterogeneity may be larger in the Davies and Lam study. Possibly, the phenotype of Davies and Lam et al. represents different aspects of general cognitive function compared to our phenotype. Although there is evidence to support ethnicity dependency of the APOE genotype on brain function, this is not likely to be an explanation for the observed differences as both the study of Davies and Lam et al. and our study only included individuals from European descent. Finally, we cannot exclude the possibility that we had limited power to detect the cross-sectional associations found in the Davies and Lam study, as we do find an association with the polygenic profile score and general cognitive function at baseline. Our study sample was considerably smaller compared to the number of participants included in the Davies and Lam study. On the other hand, our phenotype was probably more homogeneous than the phenotype of Davies and Lam et al., but this may not have outweighed the smaller number of observations.

The SNP with the lowest *p*-value (but not statistically significant after adjustment for multiple testing) associated with general cognitive function at baseline was rs2782653 (*p*-value = 2 × 10^−4^, beta = −0.16) located at chr:bp 1:43950265. The C allele was associated with a lower general cognitive function at baseline, which was similar to the effect found in the Davies and Lam study. Rs2782653 was previously found to be associated with lower attained education level in the UK Biobank [43, 44]. A sensitivity analysis, in which we adjusted for education level, did not change our results.

To verify a possible effect of selection bias in the longitudinal analyses, we studied the participation rate per APOE haplotype group for each time point and did not observe a selection bias (Supplementary Table 4). Our result for the longitudinal association between rs429358 in the APOE coding region and cognitive decline is in line with the three GWASs on cognitive decline [15–17]. De Jager et al. [16] identified rs4420638, another SNP at the APOE locus which is in strong LD (*r*^2^ = 0.7) with rs429358 [45]. Davies et al. [15] also found rs429358 to be significantly associated with cognitive decline. Possibly we did not find a cross-sectional association between general cognitive function and the APOE SNPs cross-sectionally because ɛ4 carriers have a higher cognitive score but also a steeper decline and consequently no large differences between the different APOE genotypes are present at middle age. An alternative explanation might very well be that cognitive decline is influenced by different mechanisms than those involved in the regulation of the level of cognitive function at a certain time point. This hypothesis is supported by our finding that the APOE locus was significantly associated with cognitive decline, but not with cognitive function at baseline. This was also found in two studies of Davies, in which the APOE locus was not associated with cognitive function [14], but was associated with cognitive decline [15]. In addition, this was confirmed in a recent study that showed that APOE ε4 status was not statistically significant associated with cognition level, but was associated with cognitive decline [46]. Another recent study showed that APOE ε4 carriers have accelerated breakdown of the blood-brain barrier (BBB) in the hippocampus and medial temporal lobe contributing to cognitive decline independent of Alzheimer’s disease pathology [47]. It could be hypothesized that APOE ε4 affects cognitive decline, but not the level of cognitive function, via breakdown of the BBB. However, early Alzheimer’s pathology, in particular amyloid plaques, could also have played a role [48–50]. Further, APOE protein expression levels in specific brain regions seem to add to the development of Alzheimer’s disease [51] and may therefore possibly also contribute to decline in general cognitive function.

In conclusion, we confirm that rs429358, and thereby the APOE locus, is significantly associated to general cognitive decline, but not to general cognitive function at baseline. Baseline general cognitive function could be influenced by other mechanisms than those involved in the regulation of general cognitive decline.

## Supplementary information

Supplementary information: Materials and Methods

Supplementary information: Figures 1-7

Supplementary information: Tables 3 and 4

Supplementary Table 1

Supplementary Table 2
