# Supplementary material for: The APOE locus is linked to decline in general cognitive function: 20-years follow-up in the Doetinchem Cohort Study
Source: Transl Psychiatry. 2022 Nov 29;12:496. doi: 10.1038/s41398-022-02258-5 (PMC9708640; doi:10.1038/s41398-022-02258-5)
Supplement: Supplementary file 1 — Supplementary information: Materials and Methods [file 41398_2022_2258_MOESM1_ESM.docx]

**Supplementary information: Materials and methods**

**General cognitive function at baseline and during follow-up**

Using the four tests, we constructed a measure of general cognitive function as described by Davies and Lam *et al.* ^1^ and Trampush *et al.* ^2^. In brief, sex, age and examination round-adjusted standardized residuals were calculated for each cognition test at each round. Next, these four adjusted test scores were combined into one general cognitive function measure using a principal component analysis. In de section below, we describe each of these steps in more detail.

Age was centered at 55 years because this was the mean age at T0 (*i.e.* baseline cognition measurement). Next, every individual cognitive test score was adjusted for sex, centered baseline age, (centered baseline age)², sex*centered baseline age, and sex*(centered baseline age)² at T0 using linear regression analyses (model 1). In model 2, each individual cognitive test score was, on top of the variables in model 1, also adjusted for the number of cognition measurements and time at T0-T20 using linear mixed models with correlated random slope and intercept. This was done to account for a potential learning effect because the neuropsychological test battery was identical in every examination round. Next, the residuals were calculated, for each participant on each test and each time point, by extracting the value based on sex and age at T0 (model 1) from the value adjusted for number of cognition measurements at T0-T20 (model 2). Subsequently, the residuals were standardized (*i.e.* creating z-scores) by using the mean and SD of T0 for all time points (*i.e.* T0-T20). The standardized residuals were checked per cognition test and per time point. Standardized residuals with a distance from the nearest quartile that was greater than 1.5 times the interquartile range (IQR) in either direction were made missing, which is in line with Trampush *et al.* ^3^. When this resulted in missing values for one or more cognition tests at T0 or in cognition measurements at only one time point, participants were excluded (n=121). Next, the Cronbach's Alpha was calculated for each time point resulting in Alpha’s between 0.65 and 0.69. The Cronbach's Alpha is a measure for internal consistency and is used in this study to test whether the different cognitive tests measure the same construct (*i.e.* general cognitive function). Dropping one of the four cognition tests did not improve the Alpha at any time point (*i.e*. T0-T20), therefore all four cognition tests were used for cognition principal component analyses (cPCAs) to construct the general cognitive function measure. The individual cPCA scores were calculated at T0. The percentage of explained variance of the first unrotated cPCA was 49.6%. Next, a linear regression model was fitted with the first unrotated cPCA as dependent variable and the standardized residuals of the four cognition tests as independent variables to obtain the estimates of these four tests at T0. Subsequently, by using the estimates of the linear regression analyses at T0 in a prediction model, the cPCA scores at T5-T20 could be calculated. Just as for calculating the z-scores, T0 was used as basis for calculating the cPCA scores. Finally, to overcome relatedness regarding first and second degree kinship (*i.e.* an identity by descent (IBD) >0.185 between pairs of individuals) participants with an IBD above the cutoff point and having the lowest call-rate were excluded (n=404) ^4^. After all the steps had been taken, also shown in Figure 2 of the manuscript, 2559 participants were left to study the association between SNPs and general cognitive function and decline.

All previous steps were performed in R with RStudio interface version 1.2.1335 ^5^ using the following packages: haven version 2.0.0, dplyr version 0.7.8, tidyr version 0.8.2, lme4 version 1.1-20, ggplot2 version 3.1.0, xlsx version 0.6.1, psych version 1.8.12, factoextra version 1.0.5 , FactoMineR version 1.41, and tibble version. 2.0.1.

**References**

1. Davies G, Lam M, Harris SE, Trampush JW, Luciano M, Hill WD *et al.* Study of 300,486 individuals identifies 148 independent genetic loci influencing general cognitive function. *Nature communications* 2018; **9**(1)**:** 2098.

2. Trampush JW, Yang ML, Yu J, Knowles E, Davies G, Liewald DC *et al.* GWAS meta-analysis reveals novel loci and genetic correlates for general cognitive function: a report from the COGENT consortium. *Molecular psychiatry* 2017; **22**(3)**:** 336-345.

3. Trampush JW, Yang MLZ, Yu J, Knowles E, Davies G, Liewald DC *et al.* GWAS meta-analysis reveals novel loci and genetic correlates for general cognitive function: a report from the COGENT consortium. *Molecular psychiatry* 2017; **22**(11)**:** 1651-1652.

4. Anderson CA, Pettersson FH, Clarke GM, Cardon LR, Morris AP, Zondervan KT. Data quality control in genetic case-control association studies. *Nature protocols* 2010; **5**(9)**:** 1564-1573.

5. RStudio Team. RStudio: Integrated Development for R. RStudio, Inc., Boston, MA URL <http://www.rstudio.com/>. 2015.
