# Supplementary material for: The APOE locus is linked to decline in general cognitive function: 20-years follow-up in the Doetinchem Cohort Study
Source: Transl Psychiatry. 2022 Nov 29;12:496. doi: 10.1038/s41398-022-02258-5 (PMC9708640; doi:10.1038/s41398-022-02258-5)
Supplement: Supplementary file 2 — Supplementary information: Figures 1-7 [file 41398_2022_2258_MOESM2_ESM.docx]

**Supplementary information: Supplementary figures 1-7**

**
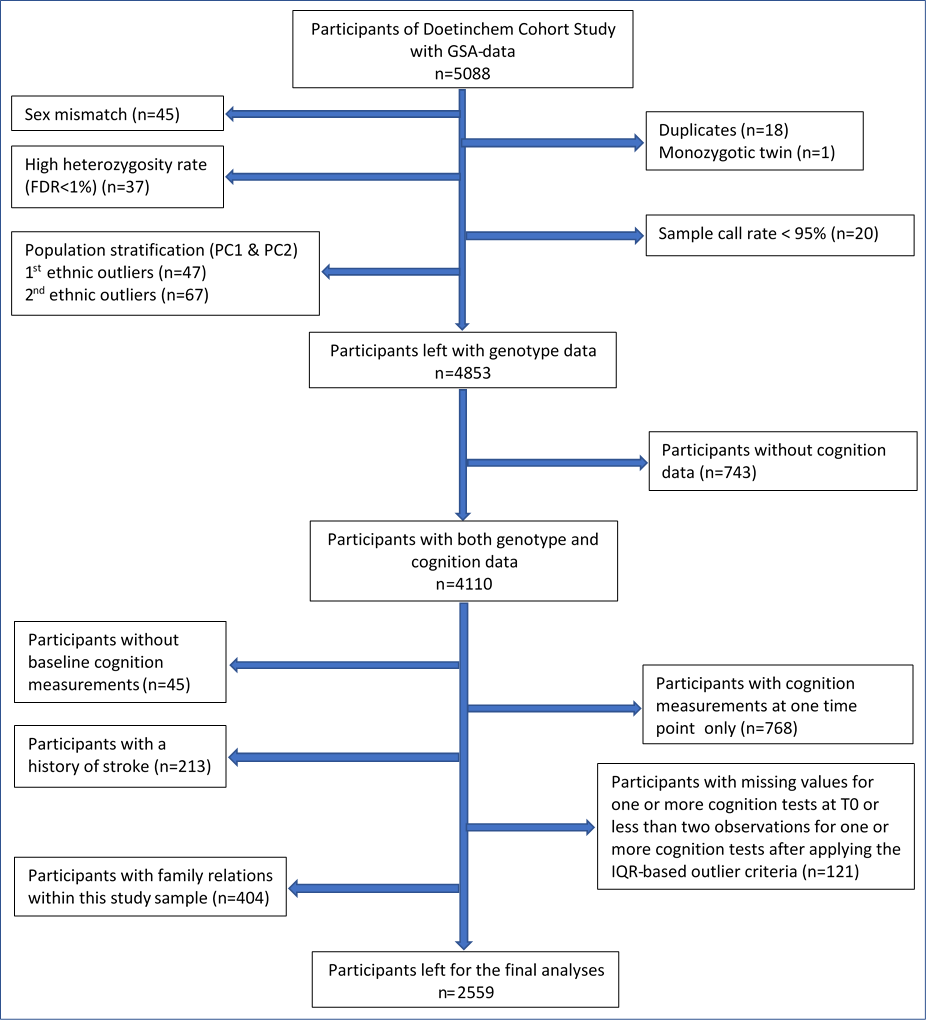
**

**Supplementary figure 1. Flowchart of quality control steps and exclusion criteria.** This figure shows the number of participants excluded in each step. The quality control steps were performed in order to obtain high quality genotype data of each participant. The exclusion criteria were performed in order to obtain high quality cognition data of each participant.

*Note:* GSA-data=Illumina Infinium Global Screening Array-24 Kit-data; FDR=false discovery rate; PC=principal component; IQR=interquartile range.


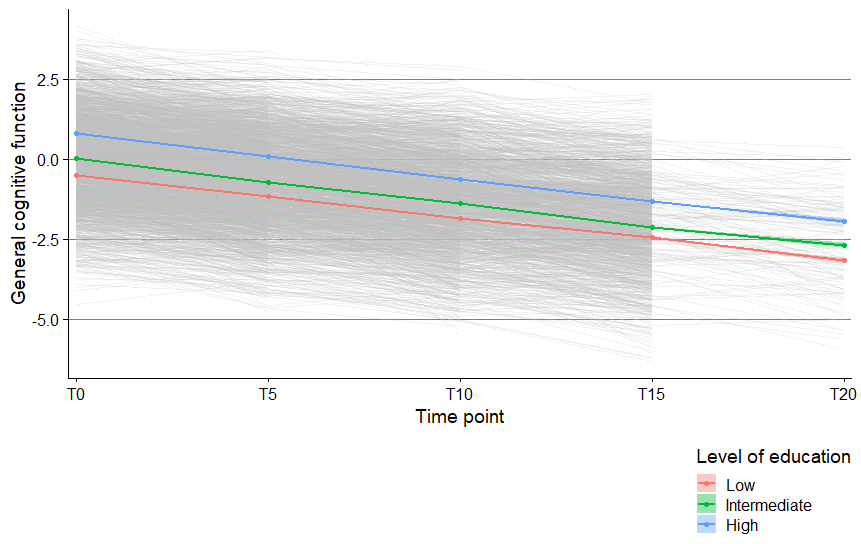


**Supplementary figure 2. Trajectories of general cognitive function stratified by education level.** This figure shows the trajectories (with 95% confidence intervals) of general cognitive function stratified by education level up to 20-years follow-up (n=2559). Education level was categorized into low (intermediate secondary education or less), intermediate (intermediate vocational and higher secondary education) and high (higher vocational education or university). T0-T20 represents time in years.

**
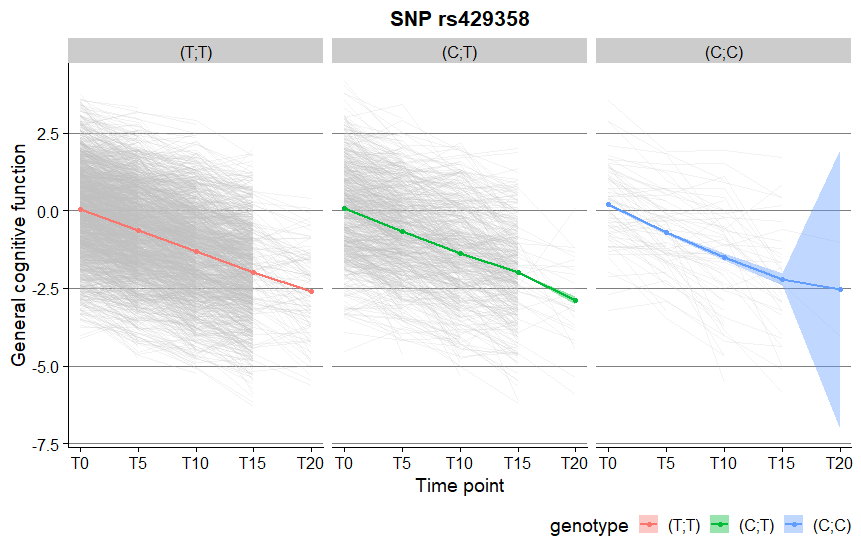
**

**Supplementary figure 3. Trajectories of general cognitive function stratified by rs429358 genotype.** This figure shows the trajectories (with 95% confidence intervals) of general cognitive function stratified by rs429358 genotype up to 20-years follow-up (n=2559). T0-T20 represents time in years.

**
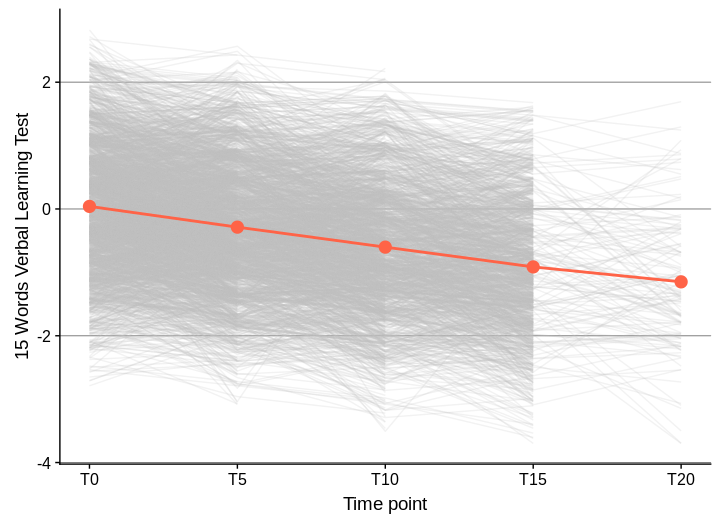
**

**Supplementary figure 4. Trajectories of the 15 Words Verbal Learning Test.** Sex, age and examination round-adjusted standardized residuals of the 15 Words Verbal Learning Test up to 20-years follow-up. T0-T20 represents time in years.

**
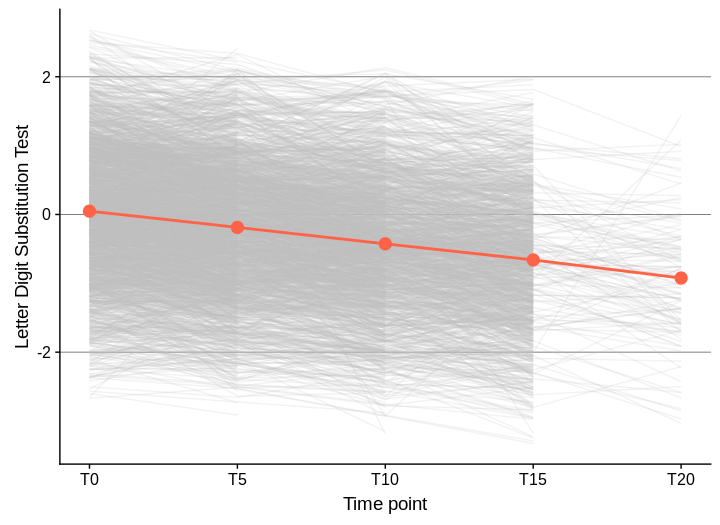
**

**Supplementary figure 5. Trajectories of the Letter Digit Substitution Test. S**ex, age and examination round-adjusted standardized residuals of the Letter Digit Substitution Test up to 20-years follow-up. T0-T20 represents time in years.

**
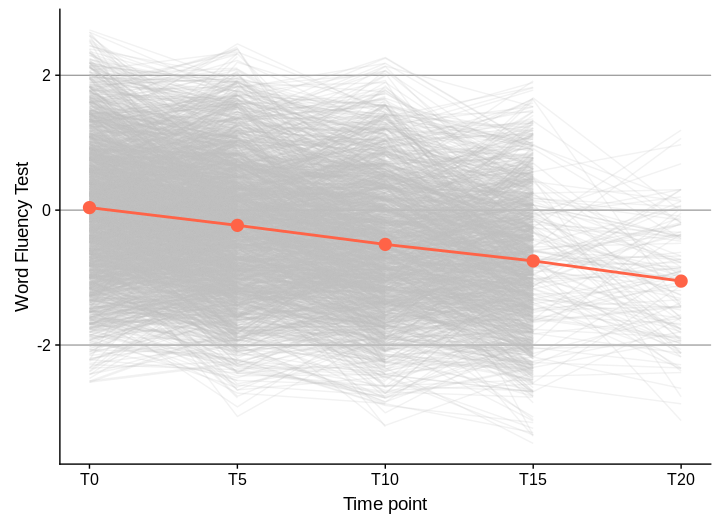
**

**Supplementary figure 6. Trajectories of the Word Fluency Test.** Sex, age and examination round-adjusted standardized residuals of the Word Fluency Test up to 20-years follow-up. T0-T20 represents time in years.

**
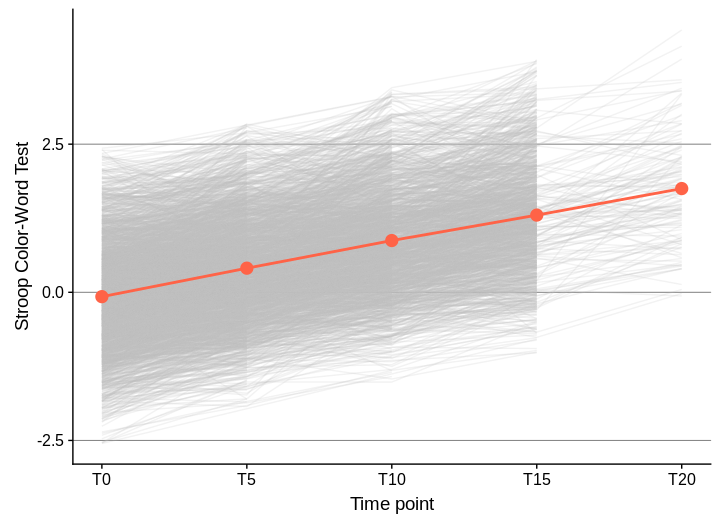
**

**Supplementary figure 7. Trajectories of the Stroop Color-Word Test.** Sex, age and examination round-adjusted standardized residuals of the Stroop Color-Word Test up to 20-years follow-up. T0-T20 represents time in years.
