# Supplementary material for: The APOE locus is linked to decline in general cognitive function: 20-years follow-up in the Doetinchem Cohort Study
Source: Transl Psychiatry. 2022 Nov 29;12:496. doi: 10.1038/s41398-022-02258-5 (PMC9708640; doi:10.1038/s41398-022-02258-5)
Supplement: Supplementary file 3 — Supplementary information: Tables 3 and 4 [file 41398_2022_2258_MOESM3_ESM.docx]

**Supplementary information: Tables 3 and 4**

**Supplementary table 3. APOE haplotypes stratified by education level.**

| **Haplotypes** | **Low** | **Middle** | **High** | **APOE groups** |
| --- | --- | --- | --- | --- |
|  | **N=1044** | **N=748** | **N=767** |  |
| ɛ2/ɛ2 | 1% | 1% | 0% | ɛ2 carriers |
| ɛ2/ɛ3 | 7% | 7% | 5% | ɛ2 carriers |
| ɛ3/ɛ2 | 5% | 7% | 6% | ɛ2 carriers |
| ɛ3/ɛ3 | 57% | 56% | 60% | ɛ3 homozygotes |
| ɛ3/ɛ4 | 11% | 11% | 11% | ɛ4 carriers |
| ɛ2/ɛ4 | 1% | 2% | 2% | ɛ4 carriers |
| ɛ4/ɛ2 | 1% | 1% | 2% | ɛ4 carriers |
| ɛ4/ɛ3 | 13% | 12% | 11% | ɛ4 carriers |
| ɛ4/ɛ4 | 3% | 2% | 3% | ɛ4 carriers |

Education level was measured as the highest level reached during follow-up and categorized into low (intermediate secondary education or less), intermediate (intermediate vocational and higher secondary education) and high (higher vocational education or university).

**Supplementary table 4. Participation rate by APOE haplotype group for each time point.**

| **Time point** | **participation rate of**  **ɛ2 carriers** | **participation rate of**  **ɛ3 homozygotes** | **participation rate of**  **ɛ4 carriers** |
| --- | --- | --- | --- |
|  | **N=339** | **N=1473** | **N=747** |
| 0 | 100% | 100% | 100% |
| 5 | 96% | 95% | 96% |
| 10 | 71% | 72% | 72% |
| 15 | 59% | 55% | 56% |
| 20* | 5% | 6% | 4% |

*The participant rate at T20 is low because the data collection is still in progress.
